# Association of financial status and the quality of life in Chinese women with recurrent ovarian cancer

**DOI:** 10.1186/s12955-017-0714-9

**Published:** 2017-07-17

**Authors:** Zhuyan Shao, Tao Zhu, Ping Zhang, Qiang Wen, Dan Li, Shihua Wang

**Affiliations:** 1 Department of Gynecologic Oncology, Zhejiang Cancer Hospital, 1 Banshan East Road, Hangzhou, Zhejiang 310022 China; 2 Department of Cancer Biology, Wake Forest School of Medicine, Winston Salem, NC USA

**Keywords:** recurrent ovarian cancer, quality of life, financial status

## Abstract

**Background:**

Very few studies have examined the association between financial status and the quality of life (QOL) of patients with specific cancers. Ovarian cancer survivors frequently suffer repeat recurrence and subsequent treatment and, as a result, a significant added financial burden. Financial burdens disproportionally affect patients of low income. This study examines the association between financial status, based upon family income and expenses, and QOL in Chinese women with recurrent ovarian cancer.

**Methods:**

We assessed baseline and follow-up (3-month) QOL of Chinese women with recurrent ovarian cancer using the European Organization for Research and Treatment of Cancer 30-Item Core Quality of Life Questionnaire (EORTC QLQ-C30), and the Quality of Life Ovarian Cancer 28 Questionnaire (QLQ-OV28). Financial status was stratified based upon self-reported disposable income. Linear or logistic regression models were applied to determine the relationship between QOL in each financial status category, and key demographic and clinical factors.

**Results:**

Among all 473 ovarian cancer patients**,** 123 of them met enrollment criteria were recruited to this study and completed baseline questionnaires; 95 of these patients completed the 3-month follow-up questionnaires. Our results showed that low financial status was significantly associated with worse QOL on all functioning domains and several symptom domains. QOL deteriorated during the follow-up. A significantly greater number of patients with low financial status experienced deteriorating QOL in several domains. Occupation and insurance type, two factors related to financial status, were significantly associated with QOL as well, but to a lesser extent. Education, recurrence interval, age and BMI were also significantly related to certain domains of QOL.

**Conclusions:**

Financial status is associated with QOL of Chinese women with recurrent ovarian cancer. These patients showed worsening QOL during active chemotherapy. Lower financial status is associated with a higher risk of deteriorating QOL in several domains.

## Background

Advances in treatments for ovarian cancer over the past 40 years have resulted in an increase in the 5-year survival rate from 21% to 46% among patients [1]. Unfortunately, the majority of ovarian cancer survivors will experience repeated recurrence and undergo multiple chemotherapies at varied intervals. Recurrent ovarian cancer patients must tolerate the fear of recurrence and of treatments and related side effects, all of which significantly impact quality of life (QOL). Improved QOL has been shown to increase survival rates and prolong survival time among ovarian cancer patients [2]. Studies in this field will aid in development of interventions and palliative efforts to maintain and improve QOL and thereby prolong survival time for ovarian cancer patients [3].

Indisputable realities for recurrent ovarian cancer patients are the huge costs for treatment of recurrent disease and possible loss of income due to the disease. Indeed, increased financial burdens for cancer patients has been extensively reported [4–9]. Three recent cross-sectional studies using data from large cohorts demonstrated the impacts of financial burdens on QOL of cancer patients [10–12]. These studies provide strong evidence of the impact of financial burden on the QOL and mental health of cancer patients.

Financial burdens disproportionally affect patients of low income. It is more appropriate to define financial burden in terms of both family income and expenses. Financial burden is influenced by local living standards. Different definitions of financial burden have been previously reported, such as length of financial reserve [10], patient charges [13], economic events [4], self-reported financial problems [12] and other definitions[11]. The lack of a uniform definition of financial burden highlights the importance of studying the impact of financial burden on QOL in different ethnic populations with different living conditions.

Few studies have examined the association of financial burden on the QOL of patients with specific cancers [4]. Our study aimed to assess the association of financial status and QOL among Chinese women actively undergoing chemotherapy for recurrent ovarian cancer.

## Methods

### Patients

This study was approved by the Ethics Committee of Zhejiang Cancer Hospital (IRB2012–21-1). All patients signed informed consent forms prior to completing the surveys.

A total of 473 ovarian cancer patients underwent either cytoreductive surgery or chemotherapy at the Department of Gynecologic Oncology of Zhejiang Cancer Hospital from June, 2012 to February, 2014. Diagnoses of recurrent ovarian cancer were based upon the criteria of the Gynecological Cancer Intergroup (GCIG): (1) A 20% increase in the sum of tumor diameters (RECIST 1.1 definition) and CA 125 ≥ 2× the upper limit of response range (ULRR) documented on two occasions (repeated measurement of CA 125 not less than 1 week after the first elevated CA 125 level); (2) Any new lesions (measurable or non-measurable); (3) unequivocal increase in non-target disease; and (4) CA 125 ≥ 2× the nadir value on two occasions [14]. A total of 123 eligible patients with newly or previously diagnosed recurrent ovarian cancer who were actively undergoing chemotherapy were enrolled in this study.

Demographic and clinical data of the enrolled participants, including age, marital status, education, occupation, type of insurance, financial status, and number of recurrences and intervals between recurrences, were collected by reviewing medical records and interviewing patients. The recently adopted “universal health care” policy allows the government to provide medical insurance to all Chinese citizens [15]. However, patients with urban medical insurance have a much lower out-of-pocket cost than those with rural medical insurance. Financial status was based on self-reported annual family income minus expenses (disposable income). Patients were divided into three financial strata based on this information: low (less than $6000); fair ($6000 to $25,000); and good (over $25,000).

### QOL evaluation

Two commonly-used questionnaires were employed to estimate the QOL of study participants: the simplified Chinese version (3.0) of the European Organization for Research and Treatment [EORCT] 30-Item Core Quality of Life Questionnaire (QLQ-C30), and the simplified Chinese version of the QLQ-OV28 questionnaire which is specific to ovarian cancer. Previous studies have shown that both questionnaires yield outcomes that demonstrate reliability, validity, feasibility, and reactivity [16]. Both the QLQ C30 and the QLQ-OV28 questionnaire were administrated at a baseline time point and again after 3–4 cycles of chemotherapy (at roughly 3 months). Scoring and scaling of questionnaires were conducted in strict accordance with the steps stipulated by the EORTC QOL group. Higher scores of functioning and global health in the QLQ-C30, and sexual functioning in the QLQ-OV28, indicated higher QOL. Higher individual symptom scores represented more severe symptoms [17, 18].

### Statistical analysis

Statistical analysis was performed using SAS (Cary, NC) version 9.3 software. Descriptive statistics were applied to demographic and clinical data, and QOL outcomes at baseline and follow-up. The difference between baseline and follow-up QOL outcomes was examined by paired T-test. The relationship between demographic and clinical factors and financial status and QOL outcomes were evaluated using univariate or multivariate linear regression models. Logistic regression models were applied to assess whether financial status could predict the risk of deteriorating QOL over time. Statistically significant results were indicated by *P*-values <0.05.

## Results

### Demographic and clinical information

Detailed information concerning age, marital status, education, occupation, financial status, insurance type, recurrences times and intervals, and sites of recurrence is shown in Table 1, along with stratification by financial status. Our results showed that while patients in different financial strata had similar age, marital status, circumstances of recurrence and chemotherapy regimens, the group of patients with higher financial status was enriched for those with higher education, urban medical insurance, self-employed status, and employees and retirees from enterprise.

**Table 1:** Demographic and clinical data based on financial status

Variables	Overall (*n* = 123)	Financial status			
		Low (*n* = 20)	Fair (*n* = 47)	Good (*n* = 56)	*P*
Age	53.4 ± 9.3	51.5 ± 10.1	53.7 ± 9.8	53.9 ± 8.6	0.585
BMI	21.1 ± 1.3	21.0 ± 1.5	21.2 ± 1.4	21.0 ± 1.1	0.737
Marital status					
Married	113 (91.9%)	17 (85.0%)	42 (89.4%)	54 (96.4%)	0.438
Single^a^	10 (8.1%)	3 (15%)	5 (10.6%)	2 (3.6%)	
Education					
Junior high school or lower	86 (78.0%)	19 (95.0%)	39 (83.0%)	38 (67.9%)	0.025
Senior high school or above	27 (22.0%)	1 (5.0%)	8 (17.0%)	18 (32.1%)	
Occupation					
Farmer or housewife	46 (37.4%)	17 (85.0%)	22 (46.8%)	7 (12.5%)	<0.0001
Factory worker	15 (12.2%)	3 (15.0%)	6 (12.8%)	6 (10.7%)	
Enterprise employee	20 (16.3%)		3 (6.4%)	17 (30.4%)	
Self-employed	7 (5.7%)		2 (4.2%)	5 (8.9%)	
Retired	35 (28.5%)		14 (29.8%)	21 (37.5%)	
Insurance type					
Urban medical insurance	75 (61.0%)	3 (15.0%)	19 (40.4%)	53 (94.6%)	<0.0001
Rural medical insurance	48 (39.0%)	17 (85.0%)	28 (59.6%)	3 (5.4%)	
Surgery	23 (18.7%)	4 (20.0%)	9 (19.1%)	10 (17.9%)	0.972
Number of recurrences					
1	80 (65.0%)	15 (75.0%)	31 (66.0%)	34 (60.7%)	0.509
2–10	43 (35.0%)	5 (25.0%)	16 (34.0%)	22 (39.3%)	
Recurrence interval					
≤ 12 months	74 (60.2%)	12 (60.0%)	27 (57.4%)	35 (62.5%)	0.451
12–90 months	49 (39.8%)	8 (40.0%)	20 (42.6%)	21 (37.5%)	
Site of recurrence					
Pelvic	27 (22.0%)	6 (30.0%)	9 (19.1%)	12 (21.4%)	0.380
Abdominal	41 (33.3%)	3 (15.0%)	19 (40.4%)	19 (33.9%)	
Distant metastasis	55 (44.7)	11 (55.0%)	19 (40.4%)	25 (44.7%)	
Chemotherapy					
Paclitaxel plus Carboplatin	36 (29.3%)	6 (30.0%)	14 (29.8%)	16 (28.6%)	0.969
Docetaxel plus Carboplatin	34 (27.6%)	5 (25.0%)	11 (23.4%)	18 (32.1%)	
Gemcitabine plus Cisplatin	33 (26.8%)	5 (25.0%)	14 (29.8%)	14 (25.0%)	
Others^b^	20 (16.3%)	4 (20.0%)	8 (17.0%)	8 (14.3%)	

^a^including unmarried, divorced or widowed

^b^chemotherapies with FOLFOX; or Cisplatin plus Topotecan, or Cisplatin plus Liposomal doxorubicin

### Financial status and QOL

All 123 patients finished both QLQ C30 and QLQ-OV28 questionnaires at the baseline. QOL scores of patients at different financial strata are shown in Table 2. At the baseline, the global health score (57.0 ± 18.1) in the QLQ-C30 was very low. The lowest score (64.2 ± 25.8) was in the area of social functioning. Financial difficulty (56.5 ± 21.9) was the most severe among symptom domains. The QLQ-OV28 sexuality score was extremely low. Of the 123 patients, 106 (86.2%) reported a complete loss of interest in sex and the remaining 17 (13.8%) reported a low interest in sex. A total of 112 (91.1%) patients reported a lack of sexual activity. Only 11 patients reported that they were sexually active, but all of them indicated that they experience little sexual enjoyment. Eight of them reported no vaginal dryness, two reported little vaginal dryness, and one reported obvious vaginal dryness.

**Table 2:** Relationship between financial status and QOL

Outcomes ^a^	Overall (*n* = 123)	Financial status			Coefficient (95%CI)^b^	*P*	Coefficient (95% CI)^c^	*P*
		Low (*n* = 20)	Fair (*n* = 47)	Good (*n* = 56)				
QLQ-C30								
Physical functioning	80.0 ± 18.9	74.3 ± 16.4	75.7 ± 27.2	85.7 ± 20.7	4.4 (0.2 ~ 8.6))	0.038	9.1 (4.0 ~ 14.2)	0.001
Role functioning	78.5 ± 26.3	71.7 ± 33.4	72.7 ± 27.2	85.7 ± 20.7	8.3 (2.0 ~ 14.6)	0.010	12.7 (4.7 ~ 20.7)	0.002
Cognitive functioning	78.6 ± 17.5	72.5 ± 22.5	75.5 ± 19.0	83.3 ± 12.7	5.9 (1.7 ~ 10.1)	0.006	5.4 (1.3 ~ 9.5)	0.005
Emotional functioning	72.4 ± 19.2	64.6 ± 21.3	68.4 ± 18.9	78.4 ± 17.0	7.6 (3.1 ~ 12.1)	0.001	3.5 (0.5 ~ 6.5)	0.028
Social functioning	64.2 ± 25.8	50.8 ± 29.4	55.7 ± 22.6	76.2 ± 21.8	14.3 (8.5 ~ 20.1)	<0.0001	13.4 (8.3 ~ 18.5)	0.001
Global health	57.0 ± 18.1	57.5 ± 23.7	52.7 ± 22.5	59.4 ± 20.7	NS		NS	
Financial difficulties	56.5 ± 21.9	93.3 ± 13.7	57.4 ± 28.4	27.4 ± 25.5	−32.4 (−38.5 ~ −26.2)	<0.0001	−30.1 (<0.0001)	
Fatigue	37.2 ± 19.6	39.4 ± 22.1	40.2 ± 20.7	33.9 ± 17.4	NS		−5.9 (−11.8 ~ −0.1)	0.048
Nausea and vomiting	8.5 ± 14.8	8.3 ± 13.8	9.9 ± 17.3	7.4 ± 13.1	NS		NS	
Pain	21.8 ± 25.0	28.3 ± 27.1	24.1 ± 24.8	17.6 ± 24.1	NS		−9.2 (−17.2 ~ −1.2)	0.024
Dyspnea	15.4 ± 21.9	16.7 ± 20.3	14.2 ± 18.1	16.1 ± 25.4	NS		NS	
Insomnia	32.2 ± 29.9	28.3 ± 27.1	34.0 ± 30.7	32.1 ± 30.5	NS		NS	
Appetite loss	18.7 ± 24.1	18.3 ± 27.5	18.3 ± 20.6	19.0 ± 26.1	NS		−8.8 (−15.6 ~ −2.0)	0.011
Constipation	19.0 ± 30.8	28.3 ± 34.7	27.7 ± 36.3	8.3 ± 19.3	−12.0 (−19.2 ~ −4.7)	0.001	−12.3 (−21.8 ~ −2.9)	0.011
Diarrhea	9.5 ± 17.3	5.0 ± 12.2	9.2 ± 16.6	11.3 ± 19.4	NS		NS	
QLQ-OV28								
Body image	48.0 ± 24.9	60.0 ± 27.8	50.4 ± 22.9	41.7 ± 24.0	−4.0 (−7.9 ~ −0.2)	0.003	−10.0 (−15.7 ~ −4.2)	0.011
Sexual functioning	3.9 ± 9.6	6.67 ± 12.6	3.5 ± 10.4	3.2 ± 7.4	NS		NS	
Attitude toward disease/treatment	60.5 ± 27.5	76.7 ± 23.6	66.7 ± 25.1	49.6 ± 26.6	- 14.3 (−20.5 ~ −8.0)	<0.0001	−12.9 (−1.2 ~ −6.3)	0.002
Abdominal symptoms	19.7 ± 16.3	22.1 ± 20.7	23.1 ± 15.7	16.1 ± 14.5	NS		NS	
Neurological symptoms	28.5 ± 22.5	30.6 ± 31.2	31.7 ± 22.5	25.0 ± 18.6	NS		NS	
Menopausal symptoms	15.1 ± 17.5	16.7 ± 12.6	12.7 ± 14.8	14.6 ± 19.3	NS		NS	
Other side-effects	15.5 ± 13.1	22.3 ± 19.6	16.3 ± 12.6	13.7 ± 11.8	−4.0 (−7.3 ~ −0.6)	0.020	−5.3 (−8.8 ~ −1.8)	0.018

^a^Higher scores of functioning and global health in the QLQ-C30, and sexual functioning in the QLQ-OV28, indicate higher QOL. A higher score of individual symptoms represents increased symptom severity

^b^Univariate linear regression analysis. CI confidence interval

^c^Multivariate analysis controlled for age, marital status, education, occupation, surgery, insurance type, number and interval of recurrence

Linear regression models were used to establish the correlation between financial status and QOL at the baseline measurement (Table 2). Our results showed that patients with low financial status fared worse with respect to physical functioning (*P* = 0.038), role functioning (*P* = 0.010), cognitive functioning (*P* = 0.005), emotional functioning (*P* = 0.006) and social functioning (*P* < 0.0001). They suffered greater financial difficulties (*P* < 0.0001), more severe symptoms of constipation (*P* = 0.001), poor body image (*P* = 0.003), poor attitude toward treatment and disease (*P* < 0.0001), and other chemotherapy side effects (*P* = 0.020). Most of these associations were maintained or became even more significant after adjusting for age, marriage, education, occupation, surgery, insurance type, and recurrence times and intervals.

There were 95 patients that completed both the baseline and follow-up surveys and changes in QOL outcomes were determined for this group. Our results showed that physical, role functioning and cognitive functioning, as well as the domains of nausea and vomiting, insomnia, appetite loss and other side-effects were deteriorated significantly (Table 3).

**Table 3:** Scores of QOL in paired 95 patients at two time points

Outcomes	Baseline	Follow-up	*P* values
QLQ-C30			
Physical functioning	77.2 ± 17.2	74.1 ± 14.8	0.039
Role functioning	75.6 ± 27.5	69.6 ± 25.5	0.047
Cognitive functioning	78.4 ± 18.2	70.9 ± 22.0	0.002
Emotional functioning	73.0 ± 18.0	75.3 ± 17.2	0.237
Social functioning	63.2 ± 27.0	58.6 ± 24.5	0.132
Global health	54.2 ± 21.7	57.0 ± 18.1	0.300
Financial difficulties	49.5 ± 33.6	51.9 ± 32.5	0.365
Fatigue	38.4 ± 19.1	37.3 ± 17.5	0.638
Nausea and vomiting	9.1 ± 15.1	15.1 ± 18.0	0.003
Pain	22.5 ± 24.2	20.0 ± 15.1	0.367
Dyspnea	15.1 ± 21.6	17.5 ± 21.1	0.320
Insomnia	34.4 ± 19.1	29.4 ± 26.2	0.001
Appetite loss	18.9 ± 23.1	26.3 ± 24.3	0.033
Constipation	20.0 ± 31.7	20.4 ± 27.6	0.912
Diarrhea	7.4 ± 13.9	6.3 ± 14.0	0.580
QLQ-OV28			
Body image	48.4 ± 24.6	45.6 ± 28.4	0.386
Sexual functioning	3.5 ± 8.7	3.0 ± 9.7	0.567
Attitude toward disease/treatment	60.4 ± 26.6	55.9 ± 27.2	0.110
Abdominal symptoms	19.6 ± 16.1	17.7 ± 14.2	0.287
Neurological symptoms	30.2 ± 22.0	31.2 ± 21.9	0.681
Menopausal symptoms	15.1 ± 17.5	17.9 ± 17.0	0.138
Other side-effects	15.5 ± 13.1	18.8 ± 13.3	0.029

We then identified patients with deteriorating QOL whose scores decreased in a functioning domain, global health and sexual functioning; or increased in other symptom domains during follow-up. We applied a logistic regression model to determine whether financial status predicted the risk of deteriorating QOL. Our results showed that patients with low financial status had a significantly higher risk of deteriorating QOL in the domains of physical functioning (*P* = 0.001), role functioning (*P* = 0.0140), emotional functioning (*P* = 0.021), pain (*P* = 0.010) and financial difficulties (*P* = 0.003) (Table 4).

**Table 4:** Relationship between financial status and deteriorating domains of QOL during chemotherapy

	Financial status			Coefficient (95% CI^a^)	
Outcomes	Low (*n* = 11)	Fair (*n* = 37)	Good (*n* = 47)		P
Physical functioning	6 (54.5%)	12 (32.4%)	6 (12.8%)	−1.2 (−2.0 ~ −0.5)	0.001
Role functioning	3 (27.3%)	14 (37.8%)	5 (10.6%)	−0.9 (−0.2 ~ 1.6)	0.014
Emotional functioning	6 (54.5%)	18 (48.6%)	13 (27.7%)	−0.7 (−1.4 ~ −0.1)	0.021
Pain	3 (27.3%)	5 (13.5%)	8 (17.0%)	−0.8 (−1.5 ~ −0.2)	0.010
Financial difficulties	4 (36.4)	11 (29.7%)	7 (14.9%)	−0.8 (−1.4 ~ −0.1)	0.003

^a^ *CI* confidence interval

### Association of other demographic and clinical factors and QOL

Linear regression analysis showed that patients with urban medical insurance demonstrated significantly increased cognitive functioning (*P* = 0.011), emotional functioning (*P* = 0.001), and social functioning (*P* = 0.0002), less severe symptoms of constipation (*P* = 0.011), financial difficulties (*P* < 0.0001), attitude toward disease and treatment (*P* = 0.001), and abdominal symptoms (*P* = 0.030). Patients’ occupations were classified as self-employed, current or retired enterprise employees with the highest incomes, factory workers with moderate incomes, and farmers and housewives with the lowest incomes. Occupations with higher income were significantly associated with better QOL in the areas of cognitive functioning (*P* = 0.040), emotional functioning (*P* = 0.002), global health (*P* < 0.0001), financial difficulties (*P* < 0.0001), and attitude toward disease and treatment (*P* = 0.044). Chemotherapy regimens were significantly associated with cognitive functioning (*P* = 0.031), nausea and vomiting (*P* = 0.045), and attitude toward disease and treatment (*P* = 0.031). Longer recurrence intervals were significantly associated with fewer financial difficulties (*P* = 0.005). Increased age was significantly associated with poor attitude toward disease and treatment (*P* = 0.014) and sexual functioning (*P* = 0.026). Patients with BMI of 20 or more suffered less severe symptoms of nausea and vomiting compared to those with BMI less than 20 (*P* = 0.003). Our study showed that marital status, and number and sites of recurrences were not associated with QOL of recurrent ovarian cancer patients. Only factors that were significantly correlated with QOL domains are shown in Table 5.

**Table 5:** Demographic and clinical factors significantly associated with QOL

	Demographic and clinical factors						
	Occupation^a^	Insurance type^b^	Education^c^	Chemotherapy	Recurrence interval^d^	Age	BMI
QLQ-C30							
Cognitive functioning	2.1 (0.040)^e^	−8.2 (0.011)		3.2 (0.031)			
Emotional functioning	3.4 (0.002)	−12.2 (0.001)					
Social functioning		−17.1 (0.0002)					
Financial difficulties	−9.3 (<0.0001)		−6.7 (0.023)		−0.8 (0.005)		
Constipation		14.4 (0.011)					
Nausea and vomiting				−2.5 (0.045)			−1.7 (0.003)
QLQ-OV28							
Attitude toward disease	−3.2 (0.044)	16.1 (0.001)		5.0 (0.031)		−0.7 (0.014)	
Sexual functioning	−1.3 (0.016)	3.8 (0.031)				−0.2 (0.026)	
Abdominal symptoms		6.5 (0.030)					
Menopausal symptoms					−0.3 (0.019)		

^a^Farmer or housewife, Factory worker, Enterprise employee, Self-employed, Retired

^b^Urban and rural medical insurance

^c^Junior high school or lower and senior high school or above

^d^≤12 months and 12–90 months

^e^coefficient (*P value*)

## Discussion

With prolonged survival, many ovarian cancer patients experience recurrence, subsequent treatments and related heavy financial burdens. Previous studies have focused on the QOL of newly diagnosed ovarian cancer patients [19–23]. To the best of our knowledge, this study represents the first investigation of the association of financial status and QOL of recurrent ovarian cancer patients.

One of the most significant findings is the strong association of financial status and QOL of recurrent ovarian cancer patients. Based on QOL scores, our data showed that the most severe symptom, indicated by the highest score, was financial difficulties. Low financial status was associated with poor QOL in all functioning domains, increased financial difficulties, and increased symptom severity. In addition, patients with low financial status experienced a higher risk of deteriorating QOL in several domains including physical, role and emotional functioning, financial difficulties and pain. In agreement with our findings, the association of financial burden and QOL has been reported in general cancer populations [10–12].

Our study revealed that both occupation and type of insurance were significantly associated with QOL. Patients with occupations of higher income were associated with better QOL in some domains. Patients with rural medical insurance which provides less coverage of medical cost and imposes more out-of-pocket expenses had lower QOL. These findings indicate that the financial status of recurrent ovarian cancer patients is associated with QOL.

Ovarian cancer recurrence creates many symptoms, such as intestinal obstruction, pain, hydrothorax, and ascites. Patients also suffer the complications caused by surgery and adverse reactions to chemotherapies. Patients with recurrent cancer tended to be more pessimistic regarding their prognoses and financial burdens than patients who were newly diagnosed. All recurrent ovarian cancer patients in our study were actively undergoing chemotherapy. Our data revealed that their QOL deteriorated in the domains of physical, role and cognitive functioning, nausea and vomiting, insomnia, appetite loss, insomnia and other side effects. This finding underscores the importance of longitudinal or dynamic assessment of QOL during the trajectory of disease [24]. Similarly, previous studies reported that ovarian cancer patients were shown to have worse QOL compared to healthy women [25], and QOL was even worse in patients with ovarian cancer at advanced stages [26].

Our study revealed that age, BMI, education, chemotherapy regimens and recurrence intervals were associated with some domains of QOL of recurrent ovarian cancer patients. Smits et al. [20, 21] reported that obese ovarian cancer patients had lower QOL in the domains of global health, physical, cognitive and social functioning, and several symptoms. Sedentary behavior was associated with lower QOL in domains of global health, physical, role, social and sexual functioning. Another study found that physical symptoms had a lower impact on global QOL than psychosocial factors in ovarian cancer patients, whereas demographic and clinical factors, such as age, cancer stage, and histology, did not have a significant impact on QOL [23]. Furthermore, global QOL and five functioning scores were reported to be dependent, in part, on age, marital status, and education in patients with gynecologic cancer [27]. The different factors associated with QOL identified in these studies underscores the importance of studying cancer patients at different stages and from different ethnic backgrounds.

Sexual dysfunction is expected in ovarian cancer patients, mainly due to the menopause caused by oophorectomy in premenopausal women [28]. Decreased hormone levels after oophorectomy result in vaginal dryness and atrophy, urinary incontinence, lower sex drive, and related symptoms. Sexual dysfunction may be also caused by distortion of self-image and fear of physical harm, as well as advanced age, poor physical status, and fatigue [29, 30]. Recurrent ovarian cancer patients and their spouses tend to focus on the cancer recurrence at the expense of sexual needs. Some patients mistakenly believed that an active sex life could lead to recurrence. Based on QLQ-OV28 scores, we found that the vast majority of patients with recurrent ovarian cancer lost sexual interest (86.2%) or had no sexual activity (91.1%). A previous study reported that <10% of early-stage ovarian cancer survivors had an interest in sex or engaged in sexual activity in spite of a comparatively high physical functioning score [31]. These findings indicate that medical intervention may be necessary to promote improvements in sexual function of ovarian cancer survivors following recurrence and subsequent treatments. Psychological counseling and careful hormone replacement therapy may be helpful.

The limitations of this study include those imposed by the non-randomized design and relatively small sample size. All patients were actively undergoing chemotherapy and were recruited from one single cancer hospital. Self-reported surveys were used to characterize financial status. No detailed expense reports were collected from patients. Other QOL related factors, such as disease progression, toxicity, mental health, family and other supports, were not analyzed in this study. Causality could not be established due to the cross sectional nature of the primary analysis. Nonetheless, this study reveals an association between financial status and QOL of Chinese women with recurrent ovarian cancer.

## Conclusions

This is the first study to investigate the association between financial status and QOL of recurrent ovarian cancer patients. These patients showed deteriorating QOL during active chemotherapy. Lower financial status is associated with a higher risk of deteriorating QOL in several domains. Our data highlight the need to draw increased attention to the financial burdens of recurrent ovarian cancer patients and the impact of this on their QOL.

## Abbreviations

CI: Confidence interval
EORTC QLQ-C30: European Organization for Research and Treatment of Cancer 30-Item Core Quality of Life Questionnaire
GCIG: Gynecological Cancer Intergroup
QLQ-OV28: Quality of Life Ovarian Cancer 28 Questionnaire
QOL: Quality of life
